# Chromatography's evolution, unlocking affinity's new solution: potential-controlled affinity membrane chromatography

**DOI:** 10.1039/d5ra08238b

**Published:** 2026-01-28

**Authors:** Tobias Steegmüller, Maeliss Nzokam, Christian Sieg, David Golonka, Nina Fridley, Sebastian P. Schwaminger, Sonja Berensmeier

**Affiliations:** a School of Engineering and Design, Technical University of Munich Boltzmannstraße 15 85748 Garching bei München Germany s.berensmeier@tum.de; b Waters | Wyatt Technology Hochstraße 12a 56307 Dernbach Germany; c NanoLab, Division of Medicinal Chemistry, Otto Loewi Research Center, Medical University of Graz Neue Stiftingtalstraße 6 8010 Graz Austria sebastian.schwaminger@medunigraz.at; d BioTechMed-Graz Mozartgasse 12 8010 Graz Austria; e Munich Institute of Integrated Materials, Energy and Process Engineering Lichtenbergstr. 4a 85748 Garching bei München Germany

## Abstract

Antibody purification is key to advancing immunotherapy, but high costs in traditional affinity chromatography remain a barrier. In response, we present a breakthrough technology for purifying antibodies from human blood plasma (HBP) and cell culture supernatant using a potential-controlled method. This innovative platform applies an electrical potential to efficiently elute up to 95% of bound antibodies from Protein A affinity membranes at optimal voltages of +2.5 to 3 V, all without the need for buffer exchange. Antibody quality, analyzed *via* Dynamic Light Scattering (DLS), Size-Exclusion Chromatography (SEC) with Multi-Angle Light Scattering (SEC–MALS), and Surface-Plasmon-Resonance (SPR), demonstrated superior retention of antibody integrity. Our potential-controlled affinity membrane chromatography (PCAMC) offers a revolutionary alternative to traditional techniques, boosting efficiency and sustainability while overcoming the limitations of conventional affinity methods. This study sets the stage for a cost-effective and eco-friendly future in antibody therapeutics.

## Abstract 2

Potential-controlled affinity membrane chromatography enables antibody purification without changes in pH or salt concentration, supporting mild processing conditions and improved antibody quality.

## Graphical abstract

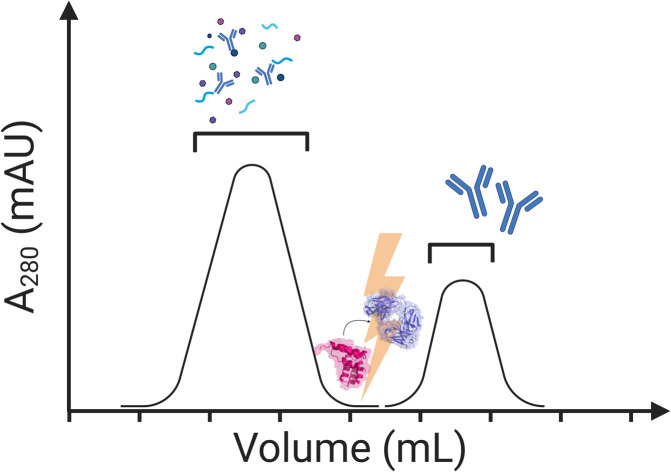

## Introduction

Ever since the COVID-19 pandemic, antibodies have gained global attention as crucial therapeutics in modern medicine. They play a pivotal role not only in medicine but also in research, various biological processes, and diagnostics. The current market size for antibodies and antibody fragments is valued at 197 billion US dollars and is expected to increase annually with a growth rate of 11.9% (*i.e.*, 2022 to 2032), driven by the growing number of therapeutics being approved^1,2^ Consequently, the efficient purification of antibodies from complex biological samples is of paramount importance to ensure their optimal functionality and purity for downstream applications. The traditional platform process for antibody purification involves Protein A (SpA) as an affinity ligand immobilized on conventional chromatographic resins, and subsequent acid elution of bound antibodies from affinity matrices.^3^ This capture step, while highly selective, effective, and used for a large antibody class, comes with significant disadvantages. The extremely low pH during elution causes a significant loss of product quality due to agglomeration,^4^ reduced column shelf life due to ligand leaching, and a heightened need for buffer exchange and regeneration, an issue that is becoming increasingly important for sustainable processing aimed at reducing chemical waste. Overall, these disadvantages make the downstream processing steps one of the driving cost factors during antibody production.^5^ Given these limitations, there is a pressing need for innovative approaches that surmount the drawbacks of conventional purification methods. Microporous membranes serve as an alternative to conventional packed-bed chromatography resins, addressing limitations like mass transfer and pressure drop.^6^ Introduced in the mid-1980s, chromatographic membranes feature through-pores without interstitial space, leading to reduced mass transfer, despite generally having lower surface areas and binding capacities compared to resin columns. These membranes allow for selective separation through adsorption/binding interactions based on ion exchange, affinity, reversed phase, and hydrophobic interactions.^7^ Affinity membrane chromatography (AMC) enhances this process by integrating functional ligands on the membranes, merging the advantages of affinity gel chromatography and membrane technology.

A key focus here is antibody purification using Protein A (SpA)-immobilized membranes. Although commercially produced (research-scale) SpA membranes are becoming more available,^8^ SpA can be immobilized onto various membrane materials through specific covalent bonding reactions. This approach allows for tailored solutions that can accommodate different geometries and material properties, providing greater flexibility beyond the constraints of commercially available options. In a previous study, we investigated the functionalization of gold sputtered membranes for affinity chromatography, establishing the process parameters for the potential controlled setup described here.^9^ Interactions between molecules and surfaces are influenced by electrostatics and environmental factors, such as salt concentration, pH, and temperature. These interactions can be manipulated in potential-controlled chromatography, where an applied electric field or surface charging is used to guide and alter the adsorption behaviour of molecules. Recent studies have explored various molecules, including proteins,^10^ DNA,^11^ and chemical compounds like maleic acids,^12,13^ focusing on how electric fields impact adsorption and desorption in separation techniques. The specific interaction of antibodies and SpA is characterized by a high affinity (*K*_a_ of 1.4 × 10^8^ M^−1^), with only the acidic elution posing challenges in regards to the process.^14^ Research on eluting affinity-bound antibodies *via* electrical potential has been limited, with notable work by Grenot and Cuilleron using electrophoretic approaches,^15^ non-affinity electrophoretic methods demonstrated by Arakawa *et al.*,^16^ and Goldstein *et al.*^17^ examining the hydrolysis effect of aqueous solutions to detach ligands by altering pH over time.^18^

This study aimed to establish the first potential-controlled elution method for affinity-bound antibodies by disrupting electrostatic interaction without the need for buffer exchange or pH adjustments. In this setup, the affinity membrane is sandwiched between two gold-sputtered polyethylene terephthalate (PET) membranes, resembling a parallel plate conductor configuration. This work is the first to focus solely on desorption from the affinity membrane positioned between electrodes, distinct from the traditional study of metallic surface desorption. We compared this potential-controlled elution approach with traditional pH-induced elution using cell culture supernatant (CCS) and human blood plasma (HBP) as model solutions. The eluted species were characterized and quantified using Size-Exclusion Chromatography (SEC) coupled with Multi-Angle Light Scattering (MALS).

## Experimental section

### Potential-controlled affinity membrane chromatography (PCAMC)

**Fig. 1:**
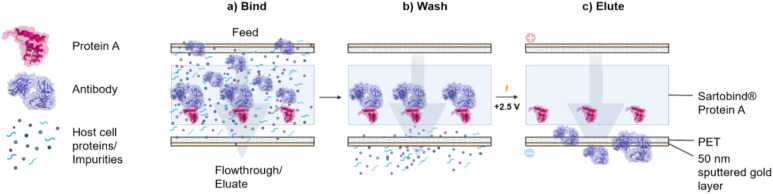
Scheme of the potential-controlled affinity membrane chromatography process (PCAMC). Abbreviations: PCAMC, potential-controlled affinity membrane chromatography; PET, polyethylene terephthalate. Created in BioRender. Steegmüller, T. (2026) https://BioRender.com/vuppn9p.

A 3D dead-end membrane holder prototype featuring a two-electrode setup was developed (Fig. S1). This design allows for scalability, as membranes could be stacked and their diameter increased. The affinity membrane matrix used in this study was a Sartobind® Protein A membrane. For the process to work, a sandwich setup was assembled with two gold-sputtered track-etched membranes surrounding the affinity membrane (Fig. 1). To assess the appropriate optimal voltage and buffer conditions to use in the PCAM process, different parameters were tested. The thermal shift assay (TSA) was used to determine the stability of antibodies in different concentrated PBS buffers and H_2_O at various pH values (SI). Likewise, to identify the optimal working voltage range, we conducted tests at various voltage levels (SI).

HBP and CCS were used as feed solutions. The sandwich setup resembles a simple parallel plate capacitor, with the track-etched gold-sputtered PET membranes acting as electrodes. Upon application of a potential, an electric field is generated. Electric contacts were glued into the holder to create contact with the 50 nm sputtered gold layer on the PET. The flow direction was through the sandwiched materials from top to bottom, exiting at the module's base. This module could be attached to an Äkta Pure FPLC system using 1/16″ fittings for the inlet and respective connectors. An FPLC ÄKTA chromatography system was used to mimic the pH process.

As analyte, 1 mg mL^−1^ monoclonal antibody (mAb) (Trastuzumab) in 1 : 1000 low-concentrated PBS buffer (lcPBS) was used. To ensure comparability between pH and potential processes, the same lcPBS as running buffer and a flow rate of 1 mL min^−1^ were used. For the pH elution process, a 50 mM sodium acetate buffer at pH 3.0 served as the elution buffer. For PCAMC, a voltage of +2.5 V was applied for the elution. A buffer exchange to 1× PBS and concentration *via* ultrafiltration (Amicon 10 kDa, Merck Germany) was performed subsequently for both pH elution and PCAMC. Elution efficiency was determined by mass balance, comparing the mass of antibody loaded onto the membrane with the mass recovered in the elution fractions. Antibody concentrations in the elution fractions were quantified by UV-vis spectroscopy at 280 nm using the Beer–Lambert law and an extinction coefficient of *ε*_(280)_ = 210 000 M^−1^ cm^−1^ for human IgG. Elution efficiency was calculated as the ratio of recovered antibody mass to the initially loaded antibody mass. Once the PCAMC process was established, we tested a more complex analyte, *i.e.*, the HBP, which contains a variety of antibodies and other proteins, including albumin and transferrin. Our goal was to achieve the purification of antibodies from complex mixtures in sufficient quantity and with adequate purity.

The PCAMC and the traditional acid elution processes were performed 3–4 times within 20–30 runs in triplicate. The resulting eluates from the PCAMC and acid elution processes were separately pooled in two final products that were used for further analyses (surface-plasmon-resonance (SPR)), dynamic light scattering (DLS), MALS, Sodium Dodecyl Sulfate–Polyacrylamide Gel Electrophoresis (SDS–PAGE).

### Determination of dynamic binding capacity (DBC_10_)

The dynamic binding capacity at 10% breakthrough (DBC_10_) was determined using increasing flow rates of 0.5, 1, 2, and 5 mL min^−1^. As analyte, a 0.1 mg mL^−1^ solution of human IgG reference mix prepared in 1× PBS was used. The IgG solution was continuously loaded onto the membrane until saturation was reached.

Measurements were conducted on membranes with increasing surface areas of 9.12 mm^2^, 16.73 mm^2^, and 24.33 mm^2^ to evaluate the effect of membrane size on binding performance. The DBC_10_ was calculated as the amount of IgG bound to the membrane at the point where 10% of the maximum signal (breakthrough curve) was reached.

### Dynamic light scattering (DLS) analysis

Measurements were performed using a Zetasizer Ultra at 25 °C in 1× PBS. The size distribution was determined for the pure buffer, the IgGmix as standard, and the antibodies after purification. The concentration, previously determined photometrically for all samples, was 0.3 mg mL^−1^. Each measurement was conducted in triplicate.

### Size-exclusion chromatography with multi-angle light scattering (SEC–MALS)

High-performance liquid chromatography (HPLC) analysis was performed using a system equipped with a degasser, quaternary pump, and multisampler. Separation was carried out on a Waters XBridge Protein Premier column (250 Å, 7.8 × 300 mm) at a flow rate of 0.75 mL min^−1^. Detection was conducted using UV absorbance at 280 nm, a DAWN® MALS detector with 18 angles, a Wyatt QELS online DLS detector at 135°, and an Optilab® differential refractive index (dRI) detector. All measurements were performed in triplicate.

### Surface-plasmon-resonance (SPR) binding site damage assessment assay

To further evaluate the impact on functionality, a qualitative SPR analysis^19^ was conducted for antibodies purified from HBP and CCS *via* pH and potential-measurements were performed in triplicate. Two binding mode sensitive regions (BMSR) on the Fc region were analysed: mAb mouse-anti-human (αHum) binding the specific CH_2_ BMSR3 of the IgG antibody, and Protein A (SpA) binding to BMSR1 on the CH_3_ position of the antibody (Fig. S2).

The functionalization of the CM5 chips was performed as follows: Channel 1 was functionalized with a mouse anti-human IgG mAb, while Channel 2 was functionalized with Protein A. Using the Amine Coupling Kit (Cytiva), we aimed to achieve a response unit (RU) value between 90 and 160. After functionalization, Channel 1 achieved an RU of 96, and Channel 2 achieved an RU of 122.

The eluted antibody species were prepared at concentrations of 12.5 and 25 µg mL^−1^. All antibody samples were diluted in HBS-P buffer. Concentration measurements were performed using UV-vis spectrophotometry at 280 nm with a nanophotometer. The concentration of antibodies capable of binding to the respective binding sites was then calculated (Fig. S3). Each measurement was conducted in triplicate to ensure accuracy and reproducibility.

## Results

Membrane chromatography (MC) setups have the advantage of minimizing mass transfer limitations, enabling higher flow rates while maintaining consistent binding capacities.^20^

**Fig. 2:**
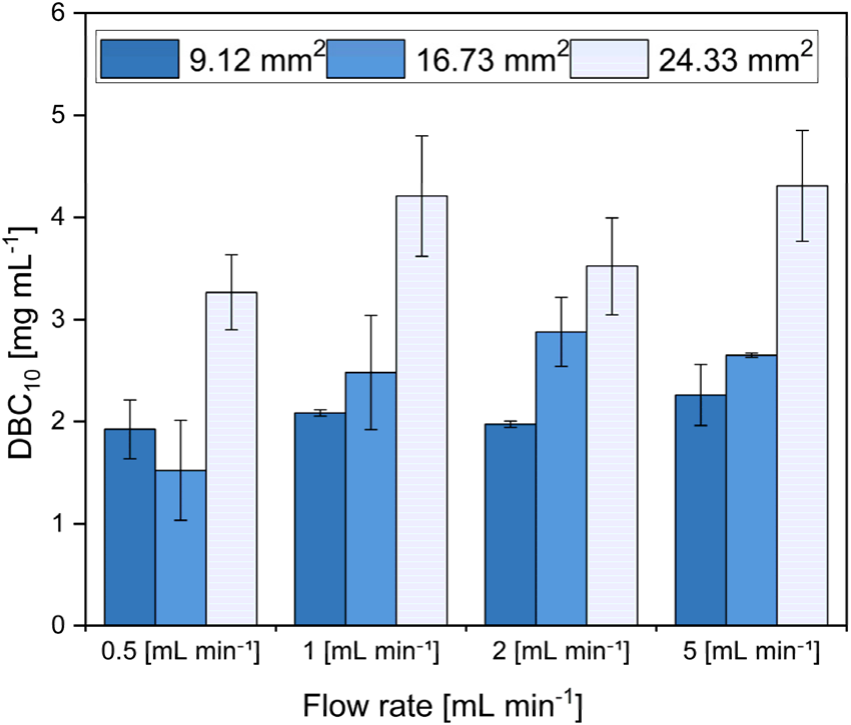
Dynamic binding capacity 10% (DBC10) at increasing flowrates for different membrane binding surface areas. The measurements were performed in triplicates (*n* = 3) and results are reported as mean ± standard deviation. Abbreviations: DBC10, dynamic binding capacity at 10% breakthrough.

This phenomenon was also observed in this study, where different flow rates and membrane surface areas were evaluated. The results showed that, due to the reduced mass transfer limitations in MC, the effect of flow rate on binding capacity was negligible (Fig. 2).

In the PCAMC set-up, higher voltages than 2.5 V damaged the electrodes, while lower voltages than 2.5 V were ineffective in removing the bound antibodies (Fig. S4). Moreover, buffers with elevated ionic strength led to a dielectric breakdown, damaging the hardware and the proteins (Fig. S5).

Therefore, a conductivity of 2.5 V was required for capacitor-like behavior, where the affinity membrane served as a dielectric medium.

1XPBS was ultimately chosen as the most appropriate binding and elution buffer for the potential experiments, as it offered stabilizing conditions and worked best with our electrical potential setup (Fig. S6A and B). H_2_O was excluded from the process as it did not function successfully with the potential setup.

**Fig. 3:**
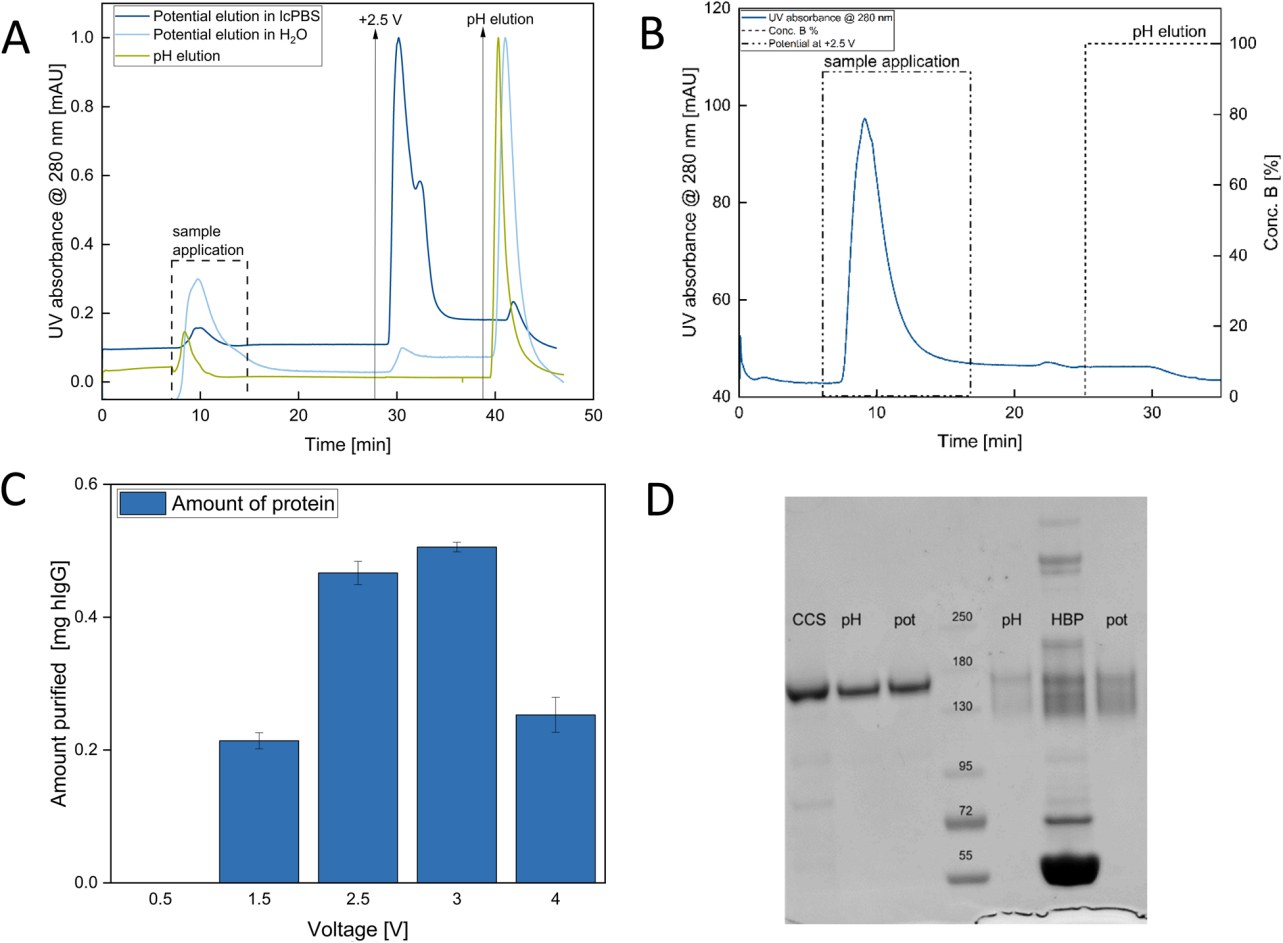
(A) Chromatogram for pH elution (green), potential elution in lcPBS (dark blue), and potential elution in Millipore H_2_O (light blue), load with 1 mg mL^−1^ Trastuzumab in lcPBS, pH elution *via* 50 mM Na-acetate pH 3.0 and potential elution at +2.5 V for 30 s, setup in the same module at 0.5 mL min^−1^; (B) chromatogram of a run with a constant applied potential of +2.5 V during the sample application and subsequent pH elution with 50 mM Na-acetate pH 3.0; (C) amount of purified antibodies at increasing elution voltages in PCAMC; (D) non reducing SDS–PAGE for cellculture supernatant (CCS) of Trastuzumab and antibodies purified from human blood plasma (HBP) each sample *via* pH elution and potential elution respectively (pH, pot). Abbreviations: CCS, culture supernatant; HBP, human blood plasma; lcPBS, low-concentrated PBS buffer; mAU, milli-absorbance units.

Tests with Millipore H_2_O (18 MΩ) showed some antibody elution following the potential switch; however, the elution efficiency was notably low, as evidenced by the absence of a pronounced elution peak in the chromatogram and by the very low antibody concentrations measured in the collected elution fractions by UV-vis spectroscopy (Fig. 3A). In contrast, application of a subsequent pH switch resulted in a distinct elution peak, indicating that the low efficiency observed with Millipore water is associated with insufficient electrostatic screening at low ionic strength. In dielectric setups, if the electric resistance of the electrolyte solution is too high, the capacitance decreases, the charging time increases, and the overall process efficiency declines.^21^ To address this issue, we used a diluted version of PBS buffer (lcPBS), resulting in a conductivity of 0.23 mS cm^−1^ (Fig. 3A). The PCAMC process resulted in over 50% decrease in the retention time from 4.06 min to 2.02 min compared to the pH shift method (Fig. 3A), where the retention time was defined as the interval between the initiation of the elution step (buffer switch) and the maximum of the elution peak in the chromatogram. This shorter cycle time enhanced overall process efficiency. Based on a single-cycle analysis, elution efficiencies of 91.2% and 74.7% were determined for the PCAMC and pH-based elution processes, respectively, based on a mass balance comparing the antibody mass loaded onto the membrane with the mass recovered in the elution fractions (Fig. 3A). Additionally, the application of potential during antibody loading completely inhibited binding (Fig. 3B). To identify the optimal elution efficiency, we tested the behavior of the antibodies at different voltages, similar to studies of elution behavior at varying pH ranges.^22^ Previous work on gold-sputtered membranes indicated no H_2_O electrolysis for cell potentials of up to 2.8 V.^23^ Here, we investigated a range of 0.5 V to 4 V. Elution voltages between +2.5 and +3.0 V showed the highest elution efficiencies of 90% and 95%, respectively, where a total of 0.5 mg of antibodies was used (Fig. 3C). SDS–PAGE analysis showed the presence of substantial aggregates in the initial HBP sample (Fig. 3D) and a purity of over 95% for each eluate.

**Fig. 4:**
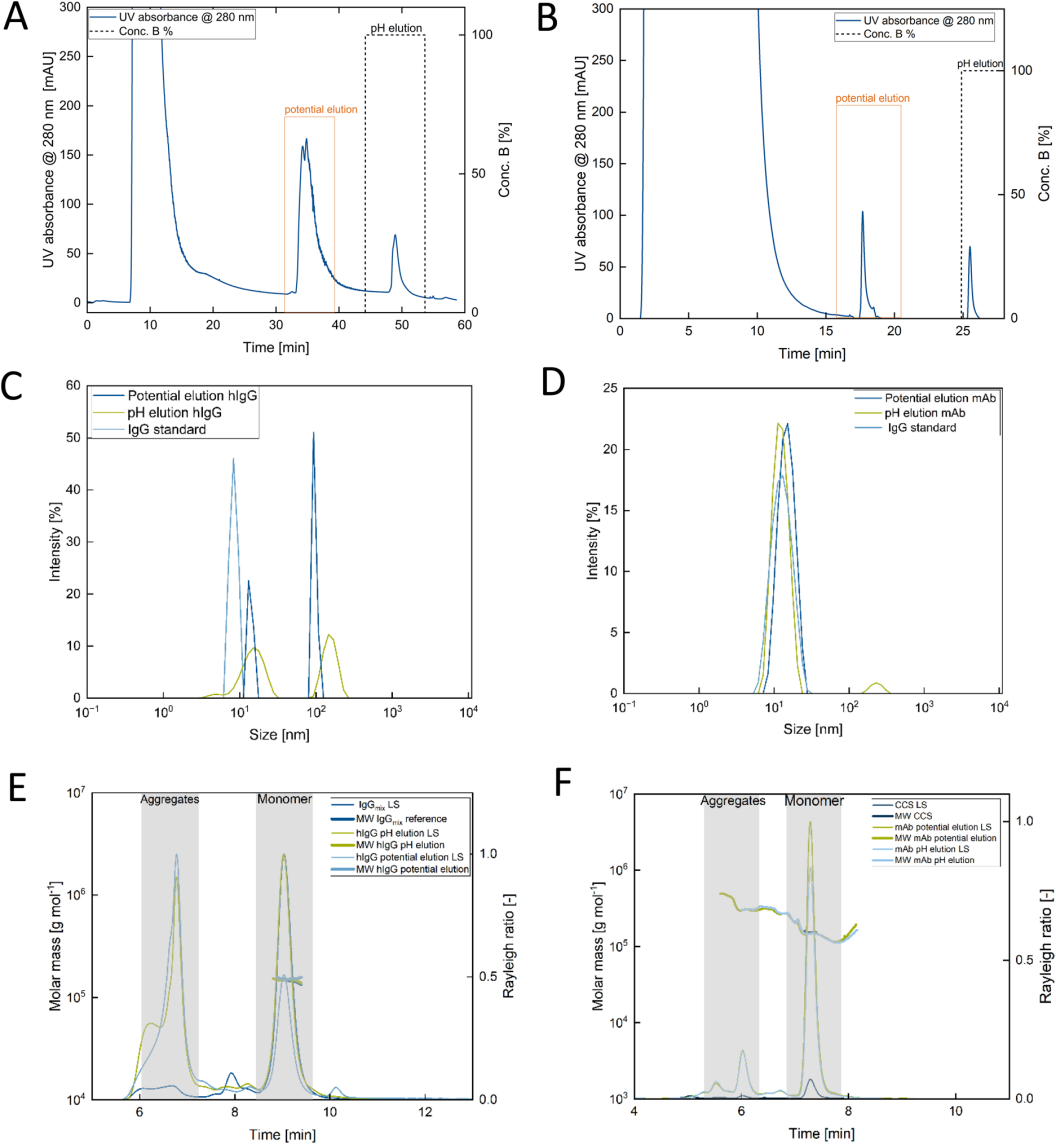
(A) Chromatogram of the PCAMC process for antibodies purified from human blood plasma with an elution at +2.5 V and subsequent pH elution with 50 mM Na-acetate at pH 3.0; (B) chromatogram of the PCAMC process for monoclonal antibodies purified from cell culture supernatant (CCS) with an elution at +2.5 V and subsequent pH elution with 50 mM Na-acetate at pH 3.0 (C) DLS measured in triplicates PCAMC *vs.* pH eluted of antibodies purified from HBP; (D) DLS measured in triplicates PCAMC *vs.* pH eluted of antibodies purified from CCS; (E) SEC–MALS PCAMC *vs.* pH of antibodies purified from HBP; (F) SEC–MALS PCAMC *vs.* pH of antibodies purified from CCS. Abbreviations: CCS, culture supernatant; MW IgG, molecular weight of Immunoglobulin G; mAB, monoclonal antibody; LS, light-scattering.

The PCAMC process successfully captured and eluted antibodies from both HBP and CCS (Fig. 4A and B).

The pH-driven elution process commonly used in the industry negatively affects the variable region of certain antibodies, leading to the formation of aggregates, which has been well documented in the literature.^24–27^ To assess potential damage and aggregation of antibodies in both the potential-driven and pH-driven elution processes, we performed DLS, SEC with SEC–MALS, and SPR analysis. In comparison to the pure hIgG reference, we observed aggregates in both HBP samples as observed in the additional population at around 100 nm in DLS (Fig. 4C). Furthermore, a shift towards a larger average size was observed for the population at around 10 nm in the HBP samples compared to the hIgG reference, which indicated the presence of impurities or higher oligomeric states. Nonetheless, DLS measurements for HBP revealed that both pH-eluted and potential-eluted samples had similar purity independent of the elution process (Fig. 4C). This aligns with our observations from the SDS–PAGE analysis, which indicated that aggregates and higher oligomeric impurities were present in the HBP sample. However, the purity levels in both the pH-eluted and potential-eluted samples were comparable. DLS results for the CCS samples also showed high similarity to the mAb chosen as reference for purity (Fig. 4D). Both the reference and potential-eluted samples were free of aggregation, while there was a small number of aggregates observed for the pH-eluted sample as seen by the additional population above 100 nm (Fig. 4D). In addition, SEC–MALS analysis was performed to overcome the limited resolution of DLS and to gain further insights into the differences between the samples. Both HBP and CCS displayed similar elution profiles with a predominant, monomeric species present for both potential-elution and pH-elution (Fig. 4E and F). For HBP, we also observed different forms of impurities, such as different oligomeric states, larger impurities, and aggregates, which was expected given the complexity of the matrix from which the antibodies were purified (Fig. 4E). The pH-eluted HBP sample exhibited a more pronounced shoulder in the aggregate region of the chromatogram, indicative of a higher number of larger aggregates generated under these conditions (Fig. 4E, green).

The potential-eluted HBP species displayed a low molecular weight (LMW) impurity after the main peak (Fig. 4E, light blue), absent in the pH-eluted HBP sample. Protein conjugate analysis revealed the absence of glycosylation for the monomeric antibodies, while the LMW impurity showed glycosylation (Fig. S7A). In addition, there was no distinct LMW population observed during the SEC–MALS analysis for the potential-eluted CCS sample compared to the pH-eluted CCS sample (Fig. 4F). Furthermore, the elution profiles during SEC–MALS for the potential-eluted sample and the pH-eluted sample were identical for CCS (Fig. 3F), and glycosylation was not affected by the type of elution process applied (Fig. S7). In SPR analysis, the choice of elution method influenced antibody quality (Table 1). The pH-eluted antibody from HBP showed impaired binding, likely due to damage at both Fc binding sites, with only 47% and 48% of hIgG able to bind to SpA and αHum, respectively. In contrast, the PCAMC process preserved antibody integrity, with 75% and 94% of hIgG binding to their respective sites (Table 1). For mAb purified from CCS, a lower difference in binding to the BMSR1 and BMSR3 sites was observed between processes, though a clear trend favouring PCAMC was noted (Table 1). The industrially used mAb, being an engineered antibody, exhibited greater pH stability compared to native hIgG from HBP, as it would have otherwise been discarded during pre-selection screenings. However, it is worth noting that PCAMC technology could avoid the discard of antibodies that can offer great therapeutic benefit but are not pH-stable. The resulting sensorgram is illustrated in Fig. S8, and the amount of antibody binding to the respective binding sites was calculated *via* the standard curve (Fig. S3).

**Table 1:** Results of the SPR binding site damage assessment assay measurements were performed in triplicates (*n* = 3) and results are reported as mean ± standard deviation. Percentage of antibody still able to bind to the respective binding site: pH elution process *versus* PCAMC^a^

Ligand	pH eluted [%]		PCAMC [%]	
	hIgG from HBP	mAb from CCS	hIgG from HBP	mAb from CCS
SpA	46.7 ± 9.0	86.7 ± 7.4	74.8 ± 2.1	85.9 ± 4.9
αHum	48.4 ± 9.2	81.3 ± 7.7	93.5 ± 0.8	96.4 ± 19.2

a Abbreviations: αHum, mouse-anti-human-Fc antibody; CCS, culture supernatant; HBP, human blood plasma; hIgG, purified human Immunoglobulin G; mAb, monoclonal antibody; PCAMC, potential-controlled affinity membrane chromatography; SpA, Protein A.

## Discussion

The PCAMC process combines the benefits of MC, affinity chromatography (AC), and potential-controlled chromatography (PCC). The FPLC ÄKTA system used to mimic the pH processenabled a clear elution of antibodies through a potential switch. Not only was elution successfully achieved, but a remarkable reduction in retention time was also observed.

Interestingly, upon application of potential during the loading of the antibody, binding was completely inhibited. This effect cannot be explained solely by a purely electrophoretic mechanism, as described in Grenot and Cuilleron,^15^ Arakawa *et al.*,^16^ and Olsen *et al.*.^28^ Similar to how a low pH (*i.e.*, environmental factors) can alter antibody–SpA interactions, the application of an electric field appears to induce electrostatic and the conformational changes in the antibodies, that preventing binding. Since the main interactions responsible for ligand-antibody binding are hydrophobic and electrostatic,^29^ the external electric field likely induces changes in the intramolecular dipoles and charge distribution within the binding pocket, thereby disrupting electrostatic interactions and facilitating the separation of bound molecules.

The absence of the aggregate species in the potential-eluted CCS sample suggests that the elution process achieved by PCAMC was slightly gentler compared to that at low pH. The higher aggregation tendency observed under low pH conditions might be related to induced conformational changes, including protein unfolding and hydrogen bond disruption.^25–27^

The presence of glycosylation in the LMW impurity of the hIgG and its absence in the protein conjugate analysis indicate that the LMW was not a fragment of the antibody of interest but rather an impurity that coeluted. In addition, since no distinct LMW population was identified for the potential-eluted CCS sample compared to the pH-eluted CCS sample, fragmentation of the antibodies during potential elution is unlikely.

Purification of antibodies from serum is particularly challenging compared to mAbs, mainly due to reduced stability, aggregation tendencies, and sample-derived contaminants that may cause ligand leakage or fouling.^30^ The PCAMC approach addresses these limitations of serum-derived hIgG by enabling efficient purification while minimizing antibody damage relative to conventional low-pH methods, as supported by biophysical analyses.

SDS–PAGE, DLS, and SEC–MALS results demonstrated a limited influence of the chosen elution method on the purity of the final product. However, DLS, MALS, and SPR analyses revealed the influence of different elution processes on product aggregation and damage. In particular, the native antibodies from HBP indicated a pronounced impact of process conditions on aggregate formation. Quantitative comparisons showed that the PCAMC process resulted in fewer large aggregates compared to the pH-based elution method. The analysis of different binding sites of the Fc-portion of both antibodies *via* SPR provide deeper insight into the damage a pH-elution process can cause. The industrially used Trastuzumab performed rather well, which is not surprising as it would not have been developed in large-scale if it was prone to acidic damage. However, the hIgG purified from HBP showed detrimental differences between the processes applied.

The PCAMC method, being a milder process, offers a promising alternative for antibodies that were previously deemed unsuitable due to their low pH instability during screening.

## Conclusion

In conclusion, we successfully confirmed our hypothesis that the PCAMC process is less damaging to antibodies. This approach resulted in lower aggregation levels and improved product quality. We are the first to demonstrate a reproducible, scalable, and reasonable approach for antibody purification using an applied external electric field. Overall, our findings suggest that the PCAMC process is gentler on the product, improves overall yield, and is especially suitable for pH-sensitive antibodies, potentially even for IgM (*via* Protein L), where acid elution leads to immediate aggregation. The whole process is scalable, cost-effective, faster, more efficient, and eco-friendly. Given today's environmental challenges, this method represents a significant step towards sustainable biotechnological processes.

## Conflicts of interest

There are no conflicts to declare.

## Supplementary Material

RA-016-D5RA08238B-s001

## Data Availability

All data included in this study are available upon request by contacting the corresponding author. See supplementary information (SI) for further data. Supplementary information is available. See DOI: https://doi.org/10.1039/d5ra08238b.
