# Supplementary material for: Chromatography's evolution, unlocking affinity's new solution: potential-controlled affinity membrane chromatography
Source: RSC Adv. 2026 Jan 28;16(7):6061–8. doi: 10.1039/d5ra08238b (PMC12850475; doi:10.1039/d5ra08238b)
Supplement: RA-016-D5RA08238B-s001 [file RA-016-D5RA08238B-s001.pdf]

Supplementary Information (SI) for RSC Advances.
This journal is © The Royal Society of Chemistry 2026

Supplemental information

Chemicals and Reagents:

Table S 1 Buffer compositions, proteins, reagents, and chemicals

Sartobind® Lab Protein A	Sartorius (Göttingen, Germany)
Track-etched 50nm gold-sputtered PET membrane with 200 nm pores	i3 Membranen (Radeberg, Germany)
10x PBS stock buffer MB-011	0.2 M KH_2PO_4, 1.5 M NaCl, 0.1% (w/v) Na-Azide, pH 7.4<br>Rockland (Limerick, PA, USA)
1x PBS (diluted from stock)	20 mM KH_2PO_4, 150 mM NaCl, 0.01% Na-Azide, pH 7.4
lcPBS (diluted from 1x PBS)	0.2 mM KH_2PO_4, 1.5 mM NaCl, pH 7.4
Na-Acetate	50 mM Na-Acetate, pH 3.0 (<br>ThermoFisher (Waltham USA)
HBS-P buffer (Hepes buffered saline with P20)	0.01 M HEPES, 0.15 M NaCl, 0.005 % v/v P20, pH 7.4<br>Cytiva (Uppsala, Sweden)
IgGmix as human IgG reference: Octagam 10%	Octapharma (Lachen, Switzerland)
Mouse-anti-human IgG CH2 (MK1A6)	Serascience (Nottingham, UK)
Protein A (recombinant SpA)	Sino Biological (Beijing, China)
Amine Coupling Kit	Cytiva (Uppsala, Sweden)
Sensor Chip CM5	Cytiva (Uppsala, Sweden)

Instrument Details:

Table S 2 List of devices and hardware

FPLC: Äkta Pure	Cytiva (Uppsala, Sweden)
Potentiostat: 1010T	Gamry (Warminster, USA)
SEC-MALS: Dawn	Waters Wyatt (Dernbach, Germany)
Nanophotometer: NP80	Implen (Munich, Germany)
DLS: Zetasizer Ultra	Malvern Panalytical (Malvern, UK)
SPR: Biacore X100	Cytiva (Uppsala, Sweden)
3D Printer: Form 4	Formlabs (Berlin, Germany)
Swinnex Filter holders 25 mm	Merck (Darmstadt, Germany)
Gold contact pin PTR Hartmann 1015-D-0.7N-AU-0.65C	Conrad Electronic (Hirschau, Germany)

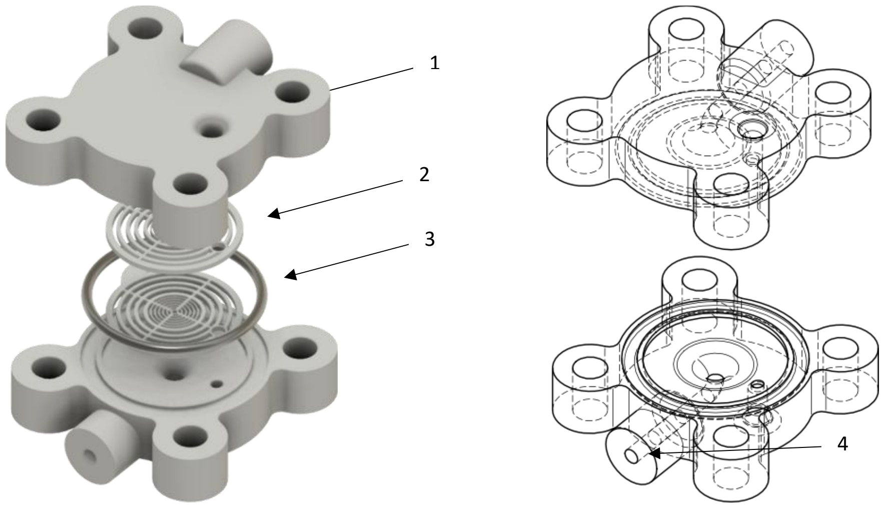

Fig. S 1 3D Setup of the PCAMC module 1) Insert point for gold pin electrode 2) Holding mesh 3) O-ring d=28 mm 4) Flow channel for liquid phase

SPR Binding site damage assessment assay:

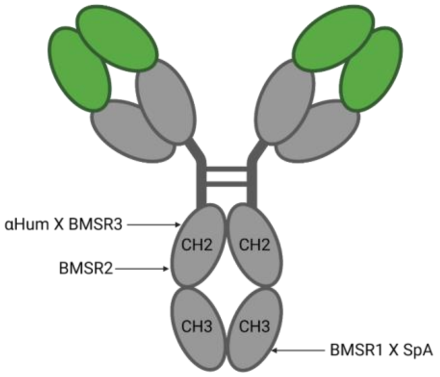

Fig. S 2 IgG antibody with respective ligand specific binding sites, the binding mode specific regions (BMSR)

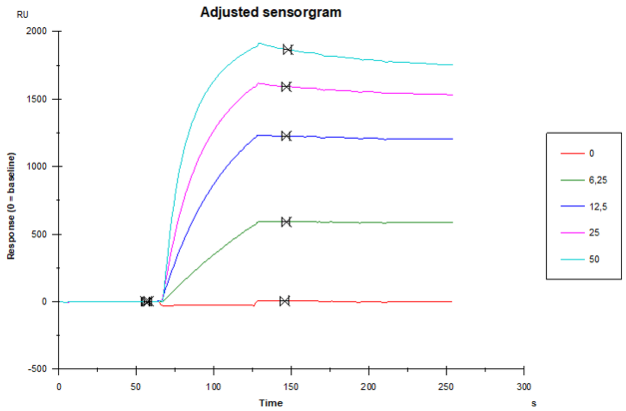

Fig. S 3 The SPR standard curve used for the calculation of bound antibodies to the respective binding sites. The concentration of antibody used in µg mL^{-1} and is given in the legend.

Experimental Setup:

Effect of voltage on antibody elution efficiency

To gain insight into the impact of voltage on the elution efficiency in the developed process, purification via potential elution was carried and varying voltages applied (+0.5 V, +1.5 V, +2.5 V, +3 V, and +4 V). This was done to assess the influence of the voltage strength on the antibody elution. The progressive increase in protein elution with ascending voltages up to +3 V remains a compelling observation. As with increasing voltages, damage to the membranes could be observed (Fig. S4), we chose +2.5 V as our optimum working range. The same flow rates and buffer conditions were used across experiments.

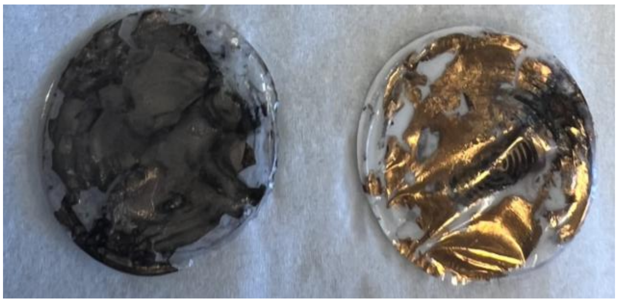

Fig. S 4 Damaged membranes after application of 5V

Thermal Shift Assay (TSA) – Determination of stability of antibodies in different PBS concentrations

The Thermal Shift Assay (TSA) was conducted to analyze the stability of the monoclonal antibody IgG1 (Trastuzumab) in PBS buffers of different concentrations and H_2O at various pH values. The protocol from Biorad was used for preparing the samples for the TSA[1]. Purified Trastuzumab was used as the analyte. Each run, in each sample was conducted in triplicates. Preliminary experiments had shown that 1x PBS (0.02M KH2PO4, 0.15M NaCl) was not suitable for the potential elution experiments as the ionic strength was too high and led to dielectric breakdown, resulting in membrane damage (Fig. S5).

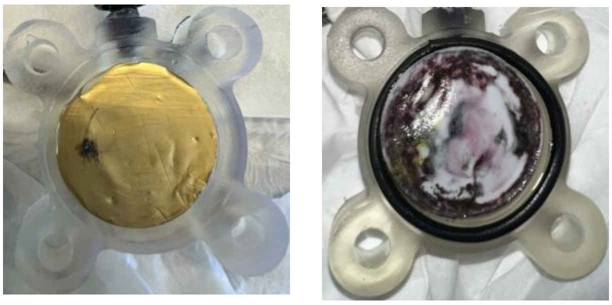

Fig. S 5 Result of high salt buffer runs: Left - burn marks on the gold sputtered membrane-gold pin point of contact due to flow of current Right side - burn marks on affinity membrane.

The results showed that the Tms (melting temperature) of the mAb was highly dependent on the buffer and pH value. It exhibited Tms ranging from 53.5 °C to 71 °C depending on the buffer and pH value. As expected, the highest measured Tm was obtained for the mAb in 1x PBS buffer (0.02 M KH_2PO_4, 0.15 M NaCl, pH 7.4) at 71 °C (Fig. S6A), which is in good agreement with literature values[2,3]. The shift in the Tm within the pH range 5 – 10 indicated that the 1x PBS buffer offered the most stabilizing medium for the antibody compared to the PBS with lower salt concentration and H_2O (Fig. S6A). However, the Tm of the mAb at a pH value of 7 in the lower concentrated PBS and H_2O at 68.5 °C and 68 °C respectively, was still within the range of reported Tms for IgG and therefore still acceptable as prospect binding buffer (Fig. S6B). Therefore, as the mAb was the most stable in the 1x PBS, a buffer exchange was conducted to ensure maximum protein stability.

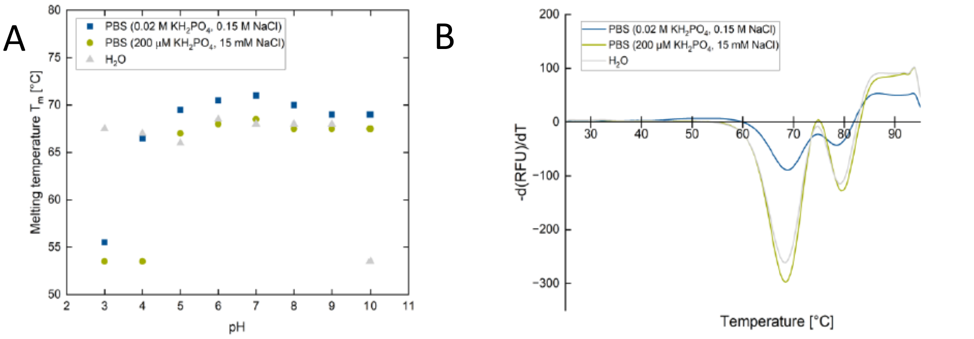

Fig. S 6 Thermal Shift Assay (TSA) of the mAb IgG_1 (Transtuzumab). A) Determined melting temperatures (TMs) in different solvents as a function of pH B) First derivative of the fluorescence emission as a function of temperature at pH 7 of the different solvents.

Kommentiert [TS1]: 146] C. B. Andersen, M. Manno, C. Rischel, M. Thorolfsson, and V. Martorana, "Aggregation of a multidomain protein: A coagulation mechanism governs aggregation of a model IgG1 antibody under weak thermal stress," *Protein Science*, vol. 19, no. 2, pp. 279–290, 2010, doi: https://doi.org/10.1002/pro.309.
[147] N. Martin, D. Ma, A. Herbet, D. Boquet, F. M. Winnik, and C. Tribet, "Prevention of Thermally Induced Aggregation of IgG Antibodies by Noncovalent Interaction with Poly(acrylate) Derivatives," *Bioma*

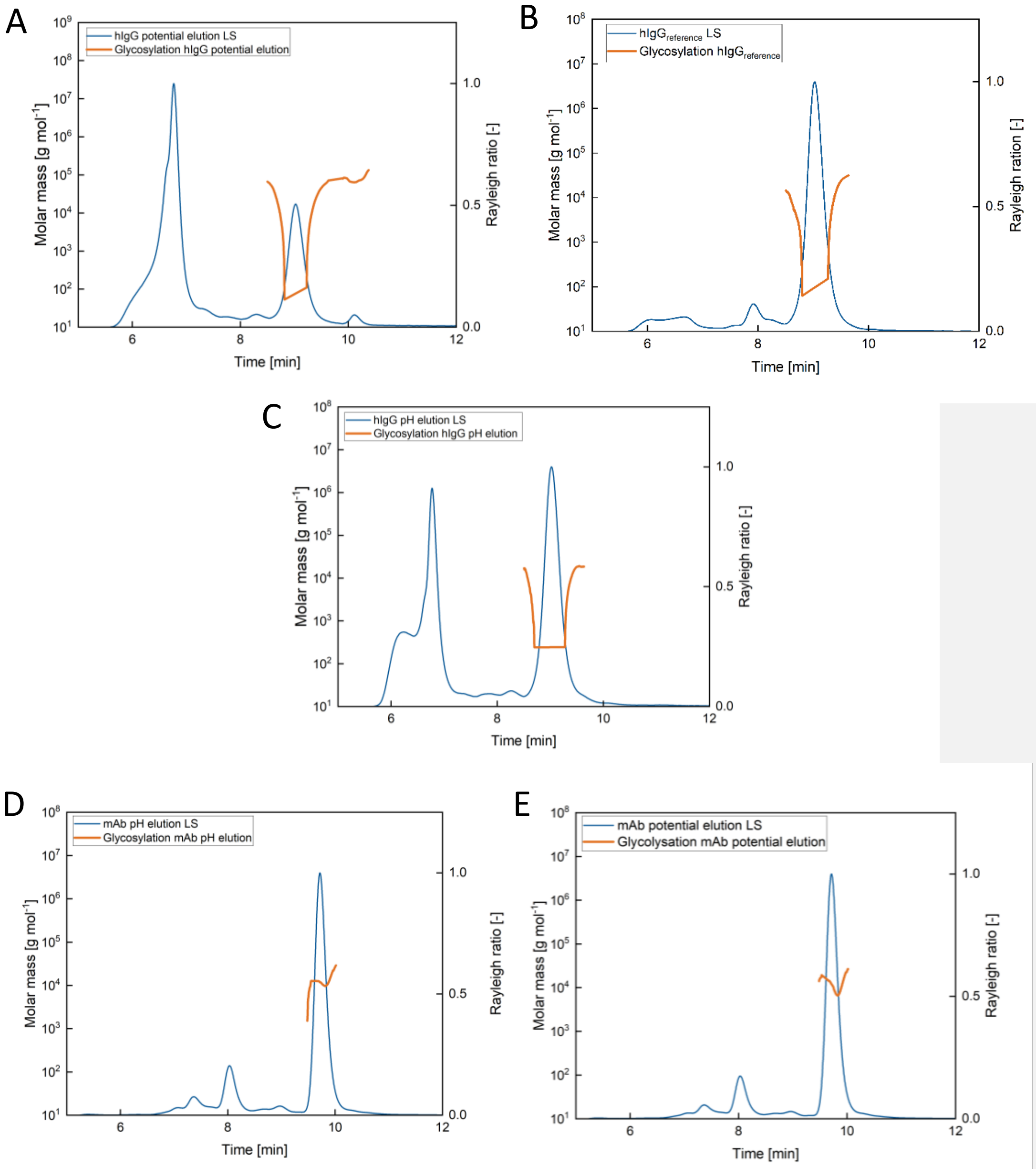

Fig. S 7 Glycosylation measurement via refractive index measurement. A) for the monomeric species and the low molecular wheight species of the antibodies purified from human blood plasma, eluted via potential; B) for the monomeric species of the human IgG reference sample; C) for the monomeric species of the antibodies purified from human blood plasma, eluted via pH shift; D) for the monomeric species of the mAb purified from cell culture supernatant, eluted via pH shift; E) for the monomeric species of the mAb purified from cell culture supernatant, eluted via potential

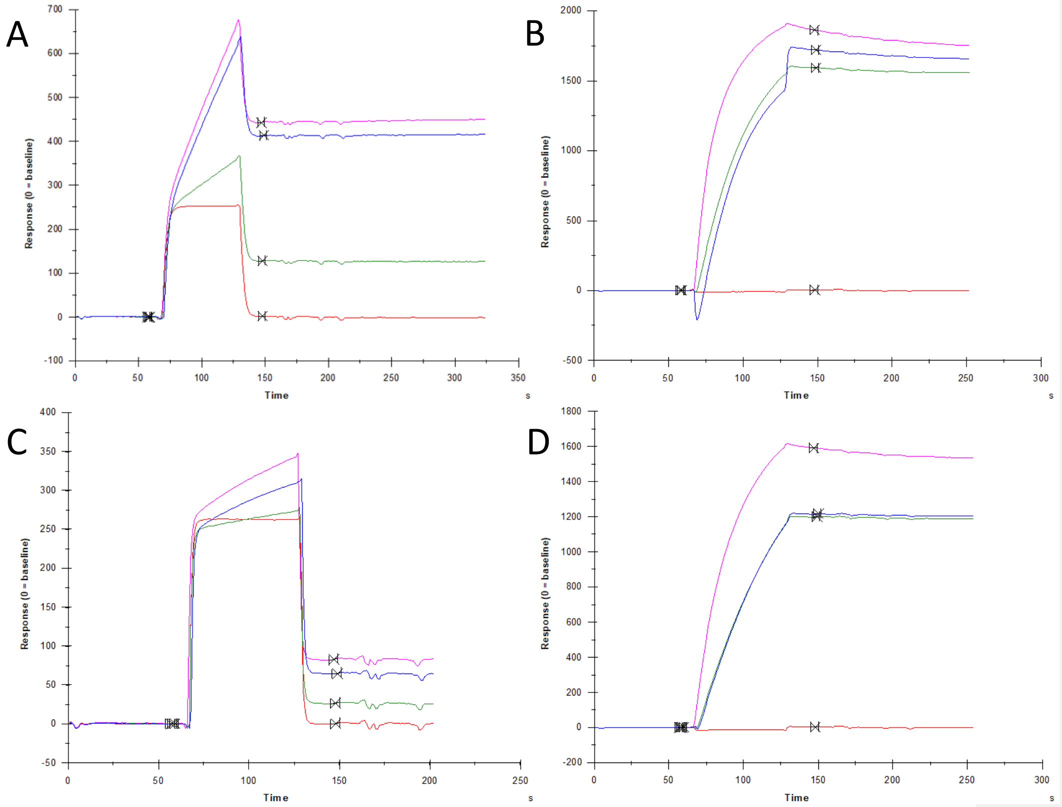

Fig. S 8 Figure for SI: SPR binding results for red: HBS-P blank buffer, green: pH eluted sample at 25 µg, blue: potential eluted sample at 25 µg, pink: standard human IgG reference at 25 µg A) antibodies purified from human blood plasma on antiHum BMSR 3; B) antibodies purified from human blood plasma on Protein A BMSR1; C) antibodies purified from cell culture supernatant on antiHum BMS3; D) antibodies purified from cellculture supernatant on Protein A BMSR1

References

1. Bio-Rad Laboratories, Inc., Protein Thermal Shift Assays Made Easy with the Protein Thermal Shift Assays Made Easy with the Bio-Rad™ Family of CFX Real-Time PCR Systems, https://www.bio-rad.com/sites/default/files/webroot/web/pdf/lsr/literature/Bulletin_7180.pdf, (accessed 8 August 2025).
2. N. Martin, D. Ma, A. Herbet, D. Boquet, F. M. Winnik and C. Tribet, Prevention of thermally induced aggregation of IgG antibodies by noncovalent interaction with poly(acrylate) derivatives, *Biomacromolecules*, 2014, **15**, 2952–2962.
3. C. B. Andersen, M. Manno, C. Rischel, M. Thórólfsson and V. Martorana, Aggregation of a multidomain protein: a coagulation mechanism governs aggregation of a model IgG1 antibody under weak thermal stress, *Protein science : a publication of the Protein Society*, 2010, **19**, 279–290.
